# Mobile health can be patient-centered and help solve inequality issues in Brazil's Unified Health System

**DOI:** 10.3389/fpubh.2023.1032412

**Published:** 2023-04-27

**Authors:** Carolina Moreira Marquez

**Affiliations:** Departamento de Medicina Preventiva da Escola Paulista de Medicina, Universidade Federal de São Paulo, São Paulo, Brazil

**Keywords:** mHealth, digital health, public health care systems, patient—centered care, SUS—Brazilian national health system

## Introduction

The use of mobile health (mHealth) is on the rise in many fields (1). The Global Observatory for eHealth of the World Health Organization defines mHealth as “medical and public health practice supported by mobile devices, such as mobile phones, patient monitoring devices, personal digital assistants, and other wireless devices (2).” The desire to overcome obstacles imposed by the pandemic made mHealth more popular than ever in the past years (3). Ethical issues, however, stimulate discussions about the theme (1, 4).

## MHealth could help the biggest governmental health care system in the world

In Brazil, one of the most challenging public health problems is attending to the universality principle of the country's Unified Health System or, in Portuguese, Sistema Único de Saúde (SUS). SUS is the world's largest health care system run by a government. The challenge is not only due to the almost 200 million users of the system, but also due to the continental dimensions of the country. Therefore, although SUS is offered to the everyone in the country, including foreigners, some areas have better access to health service than others. But, in face of that problem, some mHealth initiatives have started to help solve this inequality issue (5).

With an estimated 6 billion users in 2022, “mobile phones have become nearly ubiquitous both at the margins and the centers of capitalism (6).” That means mobile phones can serve as instruments to help solve the problem of inequality in public healthcare access worldwide, since many studies have proven mHealth can be an efficient way to deliver health services at lower costs (1, 2, 4, 5). In fact, The Universal Declaration on Bioethics and Human Rights presented by the United Nations Educational, Scientific, and Cultural Organization (UNESCO) has stated the necessity to rapidly share new therapeutic modalities or products stemming from research with countries in the developing world (7).

## Mhealth can be personalized and patient-centered too

In spite of its potential, mHealth assistance carries disadvantages over in-person care. It is no secret that mHealth deprives patients and healthcare professionals (HCP) of essential elements of interpersonal communication (e.g., full body language) (4). But it also has advantages. Having the chance to communicate with the HCP and access other health services using a mobile phone in the comfort of home has proven to be cost-effective and to have, in many cases, satisfactory outcomes in patient self-efficacy, quality of relationship between HCP and patient and overall health outcomes (1, 4, 6, 7). While mobile health industry may inadvertently convey the idea that HCP can be replaced by artificial intelligence, nothing is equivalent to or better than the bond between HCP and patients. Unless a mobile application is facilitating that relationship, it is probably not as good as it could be if it created a space for that rapport to flourish.

Many studies have demonstrated patient-centered care has positive outcomes in satisfaction and self-management (8). The vulnerability of a human being in need of help is not better covered by artificial intelligence than by another human being's sensitivity. Studies have shown technology works at best when it facilitates and strengthens the relationship between the parts involved in healthcare. A meaningful relationship in the context is not only important for patients, but for HCP as well, being recently rated as the most significant source of professional satisfaction among physicians (4). Since mHealth can serve as a channel of relationship *via* synchronized and unsynchronized communication, it can serve as an environment for humanized patient-centered healthcare. Adjusting to users' preferences and enabling a meaningful experience for both sides can help in the context. Instead of rapidness and depersonalization, which characteristics associated to mobile applications, mHealth can provide unhurried, deep healthcare experiences.

## Discussion

The field of mHealth may offer promising tools against health inequalities in Brazil and other parts of the developing world. They may have quality problems, as in person healthcare does. But what determines whether healthcare is patient-centered is the focus on the patient and his or her needs and not the environment where it occurs. Different environments, including those mediated by technology, can serve as scenarios where human dignity is promoted. In fact, many solutions in mHealth have good outcomes, equivalent to those in-person (1, 2, 4–6). Therefore, due to its vast capillarity, mHealth may help attenuate the problem of inequality in healthcare assistance in public health in Brazil and other parts of the developing world and should be the target of substantial investment and research.

## Author contributions

The author confirms being the sole contributor of this work and has approved it for publication.

## References

[1] Torous J Roberts LW. The ethical use of mobile health technology in clinical psychiatry. J Nerv Ment Dis. [2017] 205:4–8. 10.1097/NMD.0000000000000596 28005647

[2] World Health Organization. mHealth: New Horizons for health Through Mobile Technologies. Geneva. Switzerland: World Health Organization (2011).

[3] Downing L. Bodies on the line: how telepsychology brought about new relationalities between therapists and their clients during the COVID-19 pandemic. J Psychosoc Res. [2021] 14:1–15. 10.1332/147867321X16291280809438

[4] Qudah B Luetsch K. The influence of mobile health applications on patient-healthcare provider relationships: a systematic, narrative review. Patient EducCouns. (2019) 102:1080–9. 10.1016/j.pec.2019.01.021 30745178

[5] Debon R Bellei EA Biduski D Volpi SS Alves AL Portella MR. Effects of using mobile health application on the health conditions of patients with arterial hypertension: a pilot trial in the context of Brazil's Family Health Strategy. Sci Rep. [2020] 10:6009. 10.1038/s41598-020-63057-w 32265476 PMC7138856

[6] Feslberger S Subramarian R. (eds.). Mobile Technology and Social Transformations: Access to Knowledge in Global Contexts. London: Routledge [2021]. p. 216.

[7] United Nations Educational Scientific Cultural Organization. Universal Declaration of Bioethics and Human Rights. [Internet]. Genebra: Unesco (2005). Available online at: https://unesdoc.unesco.org/ark:/48223/pf0000146180 (accessed April 18, 2023).

[8] Rathert C Wyrwich MD Boren SA. Patient-centered care and outcomes: a systematic review of the literature. Med Care Res Rev. [2013] 13:351–79. 10.1177/1077558712465774 23169897

